# Non-Pharmacological Nursing Interventions to Prevent Delirium in ICU Patients—An Umbrella Review with Implications for Evidence-Based Practice

**DOI:** 10.3390/jpm12050760

**Published:** 2022-05-07

**Authors:** Sandra Lange, Wioletta Mędrzycka-Dąbrowska, Adriano Friganovic, Ber Oomen, Sabina Krupa

**Affiliations:** 1 Department of Internal and Pediatric Nursing, Medical University of Gdańsk, Dębinki 7, 80-211 Gdańsk, Poland; langa94@gumed.edu.pl; 2 Department of Anesthesiology Nursing & Intensive Care, Faculty of Health Sciences, Medical University of Gdansk, 80-211 Gdańsk, Poland; 3 Department of Anesthesiology and Intensive Medicine, University Hospital Centre Zagreb, 10000 Zagreb, Croatia; adriano@hdmsarist.hr; 4 University of Applied Health Sciences, Mlinarska cesta 38, 10000 Zagreb, Croatia; 5 ESNO, European Specialist Nurses Organization, 6821 HR Arnhem, The Netherlands; secretariat@esno.org; 6 Institute of Health Sciences, College of Medical Sciences, University of Rzeszow, 35-310 Rzeszow, Poland; sabinakrupa@o2.pl

**Keywords:** non-pharmacological interventions, delirium, ICU, systematic review

## Abstract

Delirium in ICU patients is a complication associated with many adverse consequences. Given the high prevalence of this complication in critically ill patients, it is essential to develop and implement an effective management protocol to prevent delirium. Given that the cause of delirium is multifactorial, non-pharmacological multicomponent interventions are promising strategies for delirium prevention. (1) Background: To identify and evaluate published systematic review on non-pharmacological nursing interventions to prevent delirium in intensive care unit patients. (2) Methods: An umbrella review guided by the Joanna Briggs Institute was utilized. Data were obtained from PubMed, Scopus, EBSCO, Web of Science, Cochrane Library, and Google Scholar. The last search was conducted on 1 May 2022. (3) Results: Fourteen reviews met the inclusion criteria. Multicomponent interventions are the most promising methods in the fight against delirium. The patient’s family is an important part of the process and should be included in the delirium prevention scheme. Light therapy can improve the patient’s circadian rhythm and thus contribute to reducing the incidence of delirium. (4) Conclusions: Non-pharmacological nursing interventions may be effective in preventing and reducing the duration of delirium in ICU patients.

## 1. Background

Delirium is defined as an acute cognitive impairment accompanied by fluctuations in mental status and altered attention and awareness [1,2]. This disorder is frequently caused by acute illness, trauma, surgery, adverse drug reactions, or drug withdrawal. The exact cause of delirium is still unclear, but the etiology is thought to be multifactorial [3]. Delirium has an adverse effect on patient outcomes, is an independent predictor of mortality, increases ICU length of stay, and causes cognitive impairment [4,5]. It is estimated that delirium affects up to 80% of patients in intensive care units [6]. One of the major determinants of delirium is old age [7]. In addition, risk factors include severity of illness, previous dementia, malnutrition, emergency surgery or trauma prior to ICU admission, mechanical ventilation, and anxiety [8,9,10]. There are also risk factors that are modifiable. These are mostly environmental variables such as lack of visible daylight, immobilization, isolation, noise, lack of information about the tasks performed, inadequate patient care by the medical staff, untreated pain, and use of some medications [9,11,12].

Evidence-based medicine (EBM) analyses have not identified an effective pharmacological intervention for the prevention and treatment of delirium [13]. Therefore, there is a need to develop a safe and effective strategy. The preferable methods are non-pharmacological interventions [14,15]. The possible benefit of melatonin and its antagonists has been reported, but clinical data are inconclusive, and this intervention needs further study [16,17,18]. Studies have identified a correlation between thiamine deficiency and delirium [19]. There is a potential benefit of thiamine supplementation on the prevalence of delirium. However, due to the small number of studies, no clear conclusions can be drawn on how and with what effect to implement the prevention and treatment of delirium with thiamine [20,21,22].

Studies on use of non-pharmacological interventions in patients in non-ICU wards have shown a reduction in the incidence of delirium [23,24,25].

### 1.1. Aim

To identify effective non-pharmacological interventions for the prevention of delirium in ICU patients and identify other potential benefits of these methods.

### 1.2. What Is Already Known about the Topic?

- Delirium is a common complication of hospitalization among ICU patients.
- It has an impact on treatment outcomes, increases mortality, and prolongs hospitalization and cognitive impairment.
- Delirium still remains undiagnosed among ICU patients.
- Patient assessment for delirium is still not common practice in all countries.

### 1.3. What This Paper Adds?

- Non-pharmacological nursing interventions can be effective in preventing and reducing the duration of delirium in ICU patients.
- Multi-component interventions have the highest efficacy.
- The family is an important part of the prevention of delirium.
- Light therapy can improve the patient’s circadian rhythm.
- Improving sleep quality may reduce the incidence of delirium.
- Medical staff should be aware of and implement the practice of delirium assessment in patients in intensive care units.

## 2. Methods

An umbrella review methodology was used to identify and evaluate published systematic reviews on non-pharmacological nursing interventions to prevent delirium in intensive care unit patients. In medical research, an umbrella review is a review of systematic reviews or meta-analyses. They can also be called review reviews, systematic review summaries, or review syntheses. Umbrella reviews are among the highest bodies of medical evidence available today [26,27]. Therefore, to answer the research question, we decided to conduct this type of review.

### 2.1. Review Questions

What are effective non-pharmacological nursing interventions to prevent delirium in intensive care unit patients?

### 2.2. Search Strategy

Two authors systematically searched the following databases: PubMed, Scopus, EBSCO, Web of Science, and Cochrane Library databases. The following keywords were used: “ICU”, “critical care”, ”critical illnesses”, “non-pharmacological interventions”, “multicomponent interventions”, “earplugs”, “noise reduction”, “eye masks”, “lighting control”, “education”, “orientation”, “cognitive therapy”, “bright light therapy”, “music therapy”, “physical therapy”, “early mobilization”, “exercise”, “delirium”, “delirium prevention”, “systematic review”. Keywords with their combinations using AND or OR were entered. All publications were examined by title and abstract to exclude irrelevant records. Second, a manual search of the Internet using Google Scholar was conducted to find additional systematic reviews. Any discrepancies were resolved through discussion with the four researchers, and at the end of the selection process, full agreement was reached on the articles to be included. Data including author (first), aim, participants, interventions, results, and findings were extracted from all eligible studies. The initial search was from inception to 20 March 2022, with a final search on 1 May 2022. The reviews were included if all the following criteria were satisfied.

### 2.3. Inclusion and Exclusion Criteria

Studies published in the English language were included. The inclusion and exclusion criteria were developed according to the PICOS criteria for including or excluding articles in the umbrella review (Table 1).

### 2.4. Data Collection

The data extraction form, based on the JBI umbrella review guidelines [28], was used, and the most important information in the studies was included. This extraction was undertaken by two reviewers independently. The information collected from the reviewers comprised the following: author (first), type of review, methodology/search strategy, and number of studies included. The results of data collection are presented in Table 2. The following data were collected from the studies included in the reviews: author (first), aim, participants, interventions, results, and findings. The results are presented in Table 4.

### 2.5. Quality Assessment

The methodology for JBI umbrella reviews was followed [28]. Two authors assessed the methodological quality of the reviews for inclusion using the JBI Critical Appraisal Checklist for Systematic Reviews and Research Syntheses, which provides a checklist with 11 criteria (Q1–Q11). Each question must be answered yes, no, uncertain, or not applicable. The results of this evaluation are presented in Table 3 [52].

## 3. Results

**Figure 1:**
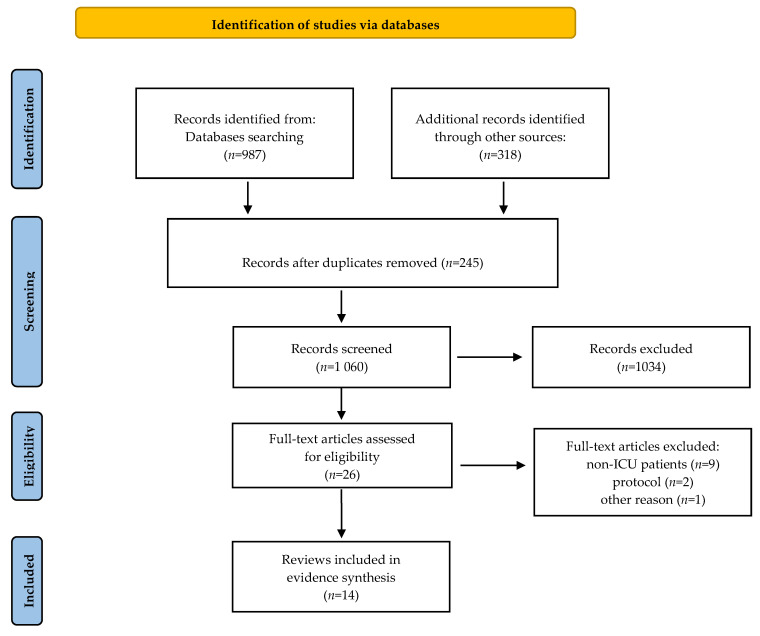
PRISMA flow diagram [38].

A total of 1305 records was initially obtained from the databases: PubMed—383, Scopus—10, EBSCO—179, Web of Science—276, Cochrane Library—139, and Google Scholar—318. After discarding duplicates and selecting titles and abstracts, 1279 were excluded, leaving 26 articles that were analyzed full text. Of these, 12 were excluded for failing to meet the inclusion criteria or the objective of the umbrella review. Fourteen reviews met the inclusion criteria [31,32,34,35,41,42,43,44,45,46,53,54]. The results are presented in Figure 1.

The review focused on non-pharmacological interventions used in ICUs for delirium. We excluded a number of reviews that analyzed pharmacological interventions or in which non-ICU patients were the study participants. However, we acknowledge that two publications reviewed studies of both pharmacological and non-pharmacological methods [35,45]. Given the clear classification of methods in these reviews, we decided to include them in our review. We only considered analyzing non-pharmacological methods. In the reviews by Luther et al. and Saritas et al. on non-pharmacological interventions, the authors included one study each using melatonin/ramelteon [32,46]. We also decided to include these reviews, excluding the melatonin/ramelteon studies.

### 3.1. Main Findings and Conclusions of the Reviews

Table 4 summarizes the main findings of the umbrella review.

### 3.2. Effects of Non-Pharmacological Nursing Interventions

Due to the presence of heterogeneous interventions in the literature, the authors decided to classify the effects of non-pharmacological nursing interventions into four main groups, which were named as follows: multicomponent non-pharmacological interventions (MLT), early mobilization (EM), family participation (FP), and environment interventions (EI). The rationale for the selection of each subtheme is presented below.

### 3.3. Multi-Component Non-Pharmacological Interventions

Luther et al., in their review, identified two non-pharmacological multicomponent interventions such as light and noise reduction and frequent patient orientation, listening to music, use of glasses, earplugs/eye covers, noise reduction, and use of natural light/darkened lighting in the evening, which were found to be effective in reducing the incidence and duration of delirium [28]. Furthermore, in the review by Kang et al., multicomponent interventions were found to be the most effective in reducing the incidence of delirium but not significant in reducing its duration. In this review, multicomponent interventions included combining some of the nine interventions or using a bundle of ABCDE [35]. The analysis in the review by Deng et al. showed that MLT was most effective in reducing the number of days of delirium and reducing ICU stay, although these results were not statistically significant. MLT was the third most effective in reducing the incidence of delirium and the second most effective in reducing in-hospital mortality [38]. The effect of multicomponent interventions in the review by Liang et al. was statistically significant in the combined analysis for the outcomes—reduction in incidence and duration of delirium, length of ward stays, and mortality [41]. According to a review by Chen et al., a multicomponent intervention consisting of seven complexes such as physical activity, family participation, cognitive stimulation, reorientation, sensory stimulation, environmental control, clinical adjustment, reorientation, sensory stimulation, environmental control, and clinical adjustment as a whole was the most effective intervention in preventing delirium in intensive care units. Interestingly, multicomponent interventions that did not include early mobilization and family participation did not show a statistically significant effect on reducing the incidence of delirium (multitreatment B: i.e., health education, reorientation, effective communication, environment control, and clinical adjustment; C: i.e., reorientation, effective communication, environment control, and clinical adjustment; D: i.e., reorientation, environment control, relaxation, and early mobilization; E: i.e., cognitive stimulation, reorientation, and family participation) [48].

### 3.4. Early Mobilization

In a review by Liang et al., seven studies analyzed the impact of early mobilization. The analysis showed positive effects in reducing the incidence (five studies) and reducing the duration of delirium (four studies). This evidence was assessed as of moderate reliability [41]. In contrast, Chen et al. found that physical activity alone did not significantly prevent delirium in the intensive care unit. However, physical activity combined with family participation had a greater effect on reducing delirium [48]. We identified one review that examined the effect of cognitive exercise on the duration of delirium in ICU patients. The meta-analysis results showed that cognitive exercise significantly reduced the incidence of delirium and the length of hospital stay in ICU patients with delirium [51].

### 3.5. Family Participation

An analysis by Qin et al. showed that family intervention was associated with a 24% lower risk of delirium and fewer days of delirium. However, it had no effect on the length of intensive care unit stay, duration of ventilation, or patient mortality [47]. One study included in a review by Bannon et al. showed a statistically significant difference in the duration of delirium between the patient’s reorientation with the voice of the family, and the voice of the unknown and the control group [31]. The analysis by Deng et al., on the other hand, found that family participation was the most effective intervention in reducing the incidence of delirium, followed by EP, MLT, CHI, PEI, and SR [38]. Of the studies in the Liang et al. review, five included family involvement. Four of these measured the incidence of delirium, and the outcome showed a significantly statistical effect on reducing the incidence of delirium (moderate confidence evidence). In addition, an analysis of three of the studies also showed a positive effect on the length of stay in the ICU, although the reliability of the evidence was assessed as very low [41]. A review by Nassar Junior et al. compared flexible and restrictive visiting policies in intensive care units. Two studies assessed the incidence of delirium in a total of 354 patients. Flexible visiting policies were associated with a lower incidence of delirium [34].

### 3.6. Environmental Interventions

In a review by Luther et al., studies relating to bright light therapy (BLT) and the use of dynamic light therapy (DLT) as single interventions showed no statistically significant differences in the incidence of delirium, although BLT therapy showed a positive effect on improving the circadian rhythm of patients [32]. In the Herling et al. review, two studies with environmental interventions (earplugs and lighting) were analyzed. In both studies, no significant differences were found with relation to the incidence of delirium [36]. A review of studies by Litton et al. found that implementation of sleep hygiene interventions, including the use of earplugs in patients admitted to the ICU, was associated with a significant reduction in the risk of delirium [30]. These outcomes agree with the review of Locihová et al. that confirm that the interventions of earplugs, eye masks, and relaxing music reduce the incidence of delirium significantly [33]. In a review by Liang et al., two studies analyzed the effect of music on the incidence of delirium. The results showed a significant effect on reducing the incidence of delirium [41].

## 4. Discussion

The incidence of delirium in ICU patients is a complication that is associated with many adverse consequences. It negatively affects not only cognitive function but also the outcomes of treatments of ICU patients and generates extremely high costs [25,51]. Due to the high incidence of this complication in critically ill patients, it is necessary to develop and implement an effective management scheme to prevent delirium [54]. Given that the cause of delirium is multifactorial, non-pharmacological multicomponent interventions are promising strategies for the prevention of delirium [55].

In the total effect analysis, non-pharmacological interventions in the review by Kanga et al. were found to be statistically significant for the onset and duration of delirium [35]. Sahawneh et al., in their integrative review, found a positive effect of non-pharmacological interventions in all eight quantitative studies, although four studies used only a single component intervention. Therefore, it can be speculated that a combination of some single interventions may have an even better effect [56]. Similarly, in the Liang et al. study, comparison of all non-pharmacological interventions, in a pooled analysis, with the control group showed a significant effect on reducing the incidence and duration of delirium and length of stay in the ICU. The reliability of this evidence was rated as low. In addition, multicomponent interventions had a higher odds ratio (OR) than single component interventions [41]. This supports the hypothesis that combining single interventions is a more effective strategy. Saritas et al. noted that all interventions from their study were effective, although not sufficient. Therefore, they also recommend the use of multicomponent methods [46]. Bannon et al. reported that although there is insufficient evidence that single or multifactorial interventions are effective, multicomponent interventions may be more reliable [31].

Herling et al. noted that physical, cognitive, and occupational therapy interventions have the potential to prevent or shorten the duration of delirium [36]. Schweitckert et al. studied the impact of early physical and occupational therapy on critically ill patients. The study showed that whole-body rehabilitation, consisting of discontinuation of sedation and physical and occupational therapy in the earliest days of critical illness, resulted in better functional outcomes at hospital discharge, shorter duration of delirium, and more ventilator-free days compared with standard care [54]. Xu et al. found a positive effect of cognitive exercise to reduce the duration of delirium and the length of hospital stay in ICU patients with delirium [51]. At the time of writing the Herling et al. review, several studies that may have influenced perceptions of early mobilization of ICU patients for delirium prevention were in progress [36,57,58]. We reached out to the authors of these studies. Unfortunately, in the final study by Wright et al., the effect of more intensive physical rehabilitation on delirium was not assessed in either primary or secondary outcomes [57]. In the review by Doiron et al., only two studies were included that reported the number of days spent in the ICU and the number of days in hospital with delirium [58]. The results of one study have already been cited [59]. In contrast, in the results of the second study, no difference was found between the groups [60]. However, the results of a study by Chen et al. showed that a multicomponent intervention that included early mobilization combined with family participation and other non-pharmacological interventions significantly reduced the incidence of delirium in the intensive care unit [48].

Single light therapy interventions have shown inconclusive results. However, a study by Engwall et al. showed the benefit of using a lighting system specifically tailored to supporting patients’ circadian rhythms on patients’ psychological well-being. Patients described bright light as healthy, pleasant, and having a positive effect on their mood and sense of security [61]. Additionally, in the review by Luther et al., the effects of MLT and light therapy (BLT and DLA) on circadian rhythm were assessed [32]. The study by Guo et al. showed a statistically-significant increase in melatonin and a decrease in cortisol in postoperative nocturnal urine levels in the MLT intervention group. These results suggest an improvement in the circadian rhythm with multicomponent interventions [62]. On the other hand, a study by Ono et al. showed better circadian cycle outcomes in the BLT treatment group [63]. The results from this review may therefore suggest that the use of these two methods in combination may increase their effectiveness in improving patients’ circadian rhythms and thus contribute to the prevention of delirium [32]. Although the Kang et al. analysis also found no significant effect of environmental interventions on the incidence and duration of delirium, it should be noted that single environmental interventions were components of multicomponent interventions [35]. Similarly, in the Herling et al. review, the study found no significant effect of earplug use or lighting on the incidence of delirium. However, it was observed that in patients sleeping with earplugs, delirium occurred later [36]. In contrast, this contradicts the results of the Litton et al., review, in which the placement of earplugs in patients admitted to the intensive care unit, either alone or as part of a sleep hygiene improvement package, was associated with a significant reduction in the risk of delirium [30]. The potential positive effect of using earplugs and eye masks on improving sleep quality and reducing the incidence of delirium was also demonstrated in a review by Locihová et al. [33].

Family involvement in the patient care process (F) in the ICU was a recent addition to the ABCDEF packet [64]. Deng et al., in their review, conducted a network meta-analysis that showed FP to be the most effective intervention in reducing the incidence of delirium, followed by EP, MLT, CHI, PEI and SR. [31]. In the Bannon et al. review, only family voice reorientation had a beneficial effect on delirium duration [36]. This suggests that the family may be an important part of the strategy to fight delirium in ICUs. Reviews of the literature by Qin et al. and Pabón-Martínez et al. confirmed that family interventions reduced the incidence of delirium [46,65]. Interventions for family participation in delirium prevention, in the Pabón-Martínez et al. scoping review, included flexible visiting hours, and direct and indirect (via audio-media) reorientation of the patient in the ICU. The study reported an association between flexible visiting and a reduction in the incidence of delirium. Other benefits of flexible visiting and patient reorientation were increased delirium-free days, reduced delirium duration, reduced incidence of infections, and reduced length of hospital stay [66]. Similarly, the review by Nassar Junior et al. found that flexible visiting policies were associated with a lower incidence of delirium. In addition, flexible visits were associated with a lower severity of anxiety symptoms among ICU patients. Involving the family in the therapeutic process had positive effects, not only for the ICU patients but also for the family itself [34]. This is consistent with a study by Kleinpell et al., which found that after implementing a project to promote and involve families in the intensive care unit, family members reported statistically significant increases in overall satisfaction, satisfaction with decision-making, and satisfaction with quality of care [67]. At the same time, we would also like to point out and agree with the researchers Chen et al. that the implementation of multifactorial interventions may put additional workloads on ICU nurses. Therefore, a multidisciplinary team should be involved in the care [48].

## 5. Conclusions

Non-pharmacological nursing interventions may be effective in preventing and shortening the duration of delirium in ICU patients. Due to the multifactorial etiology of delirium, multicomponent non-pharmacological interventions are the most promising methods. Moreover, they have shown the highest efficacy in many studies. The patient’s family is an important part of delirium prevention and should be involved in the therapeutic process. An additional benefit of including the family is to improve the families’ perceptions of the work of the medical staff. Light therapy may improve the patient’s circadian rhythm and thus reduce the incidence of delirium.

The most desirable aspect of patient-centered care for delirium is risk minimization and prevention. Medical staff should be aware of and implement delirium assessment practices and methods to minimize the risk of delirium in intensive care unit patients.

## 6. Implications for Practice

Non-pharmacological multifactorial interventions should be implemented in clinical practice in a scheme to prevent delirium in the ICU. Early mobilization, cognitive exercise, and rehabilitation of the whole body—physical therapy, occupational therapy, early movement, and transfer from bed to chair—can have positive effects. Regarding family involvement, we recommend introducing delirium education projects for the family, an extended visitation model, and acoustic reorientation developed by a family member. Through small activities such as orienting the patient to the date, place, and space; discussing current family events; and providing assistive devices that the patient uses every day (hearing aid, glasses), the family can stimulate cognitive, orientation, and memory processes. Single light therapy interventions, although not showing clear results, in combination with other interventions, e.g., noise reduction, use of music, eye masks, and ear plugs, can show beneficial results and support the circadian rhythm of patients. Care focused on delirium prevention should include the involvement of a multidisciplinary team including nurses, doctors, physiotherapists, psychologists, and occupational therapists.

## 7. Implications for Future Research

The studies that were included in the reviews mainly focused on the effect of non-pharmacological interventions on outcomes such as incidence, duration of delirium, length of hospital stay, and mortality. We suggest that future studies should also consider the impact of non-pharmacological interventions on patients’ short- and long-term cognitive function outcomes. Multifactorial interventions have been shown to be effective in reducing the incidence of delirium, but it is not always clear which combination of interventions led to the effect. Future studies should clearly specify which single interventions were combined. In addition, we suggest that future research should focus on combining single interventions, which have shown potential benefits against delirium, e.g., bright light therapy, into multicomponent interventions.

## Figures and Tables

**Table 1:** PICO criteria used to develop the research question and include or exclude studies.

PICO	Inclusion Criteria	Exclusion Criteria	Keywords	Search Strategies
Patients	Adults (>18 years), ICU patients	Adults patients of other units, children, ICU children	ICU, critical care, critical illnesses	ICU OR critical care OR critical illnesses
Interventions	Non-pharmacological interventions	Pharmacological interventions, mixed interventions, interventions only focusing on screening delirium	Non-pharmacological interventions, multicomponent interventions, earplugs, noise reduction, eye masks, lighting control, education, orientation, cognitive therapy, bright light therapy, music therapy, physical therapy, early mobilization, exercise	Non-pharmacological interventions OR multicomponent interventions OR earplugs OR noise reduction OR eye masks OR lighting control OR education OR orientation OR cognitive therapy OR bright light therapy OR music therapy OR physical therapy OR early mobilization OR exercise
Comparison	Usual care, any comparator or including no comparator	n/a	Delirium, delirium prevention	Delirium OR delirium prevention
Outcomes	Delirium-related data (e.g., reducing the incidence of delirium, shortening the duration of delirium)	n/a	n/a	n/a
Study design	Systematic review	Other types of reviews	Systematic review	Systematic review

n/a—not applicable.

**Table 2:** Results of data collection.

Author (First)	Type of Review	Methodology/Search Strategy	Number of Studies Included	In-Or Excluded (Comment)
Zhang, H. [29]	A systematic review and meta-analysis	Literature searches: MEDLINE, EMBASE, CINAHL, Cochrane Library, reference lists, “Google Scholar”. Type of studies: RCTs. Time: before August 2012	38	Excluded—No ICU patients
Rivosecch, R.M. [25]	An evidence-based systematic review	Literature searches: MEDLINE and EMBASE. Type of studies: RCTs, prospective RCTs, CCTs. Time: from 1946 to 15 October 2013	17	Excluded—Not only ICU patients
Litton, E. [30]	A systematic review and meta-analysis	Literature searches: MEDLINE, EMBASE, the Cochrane Central Register of controlled trials. Type of studies: Interventional studies. Time: period between 1966 and May 2015	9	Included
Bannon, L. [31]	A systematic review of quantitative and qualitative research	Literature searches: MEDLINE, EMBASE, CINAHL, Web of Science, AMED, PsycINFO, Cochrane Library. Type of studies: RCTs, CCTs. Time: n/d	n/d	Excluded—Protocol
Martinez, F. [23]	A systematic review and meta-analysis	Literature searches: PubMed/MEDLINE, EMBASE, PsycINFO, CINAHL, Cochrane Library, CENTRAL, LILACS, SciELO, grey literature Type of studies: Randomized trials. Time: from inception to 31 December 2012.	7	Excluded—No ICU patients
Luther, R. [32]	A systematic review of quantitative studies	Literature searches: Academic Search Complete, CINAHL Plus with Full Text, E-Journals, MEDLINE Complete, PsycARTICLES, PsycINFO. Type of studies: RCTs, and a cohort-based design. Time: 2006–2016	6	Included—Without melatonin study
Locihová, H. [33]	A systematic review	Literature searches: CINAHL, PubMed, SCOPUS. Type of studies: RCTs, CCTs. Time: 1990–2015	19	Included
Nassar Junior, A.P. [34]	A systematic review and meta-analysis	Literature searches: Medline, Scopus, Web of Science. Type of studies: Observational and randomized studies. Time:	16	Included
Kang, J. [35]	A systematic review and meta-analysis	Literature searches: MEDLINE, Cochrane Library, CINAHL, PsycINFO, EMBASE. Type of studies: cohort studies, RCTs, CBA, and CCT Time: between 2007 and 2016.	35	Included
Herling, S.F. [36]	Review	Literature searches: ENTRAL, MEDLINE, Embase, BIOSIS, International Web of Science, Latin American Caribbean Health Sciences Literature, CINAHL. Type of studies: RCTs. Time: from 1980 to 11 April 2018	12 (4 non-pharmacological interventions)	Included—Only non-pharmacological interventions analyzed
Bannon, L. [31]	A systematic review and meta-analysis	Literature searches: MEDLINE, EMBASE, CINAHL, Web of Science, PsycINFO, AMED, Cochrane Library. Type of studies: RCTs. Time: up to March 2018	15	Included
Janssen, T.L. [37]	A systematic review and meta-analysis	Literature searches: PubMed (Medline OvidSP), Embase, Cochrane Centre, Web of Science. Type of studies: RCTs, CBA. Time: in March 2018	35	Excluded—No ICU patients
Deng, L. [38]	A systematic review and network meta-analysis	Literature searches: PubMed, Embase, CINAHL, Cochrane Library. Type of studies: RCTs and cohort studies. Time: the end of June 2019	26	Included
León-Salas, B. [39]	A systematic review with meta-analysis	Literature searches: MEDLINE, EMBASE, Web of Science, Cochrane Central Register of Controlled Trials. Type of studies: RCTs. Time: 2015 to March 2019.	49	Excluded—Not only ICU patients
Ludolph, P. [40]	A systematic review	Literature searches: PubMed and CENTRAL. Type of studies: RCTs and cluster RCTs. Time: without any time constraints	8	Excluded—Not only ICU patients
Liang, S. [41]	A systematic review and meta-analysis	Literature searches: MEDLINE, CINAHL, EMBASE, Cochrane CENTRAL, Web of Science, PsycINFO, Chinese electronic databases. Type of studies: RCTs, CCTs, CBA. Time: until September 2020	34	Included
Ekeozor, C.U. [42]	A systematic review and meta-analysis	Literature searches: MEDLINE, EMBASE, PsycINFO, OpenGrey, Web of Science, reference lists of journals. Type of studies: RCTs, observational studies, and non-randomized CTs. Time: from inception to 12 February 2020	59	Excluded—No ICU patients
de Foubert, M. [43]	A systematic review	Literature searches: CINAHL, MEDLINE, EMBASE, Cochrane Library, Google Scholar, BMJ quality reports. Type of studies: randomized and quasi-experimental designs. Time: from January 2009 to February 2020.	18	Excluded—No ICU patients
Lee, Y. [44]	A systematic review of randomized controlled trials	Literature searches: PubMed, CINAHL, Embase, Cochrane Central Register of Randomized Controlled Trials. Type of studies: prospective RCTs. Time: up to 27 January 2021	9	Excluded—Not only ICU patients
Burry, L.D. [45]	A systematic review and network meta-analysis	Literature searches: MEDLINE, Embase, PsycINFO, CINAHL, Web of Science, Cochrane Library, Prospero, WHO international clinical trial. Type of studies: RCTs. Time: from inception to 8 April 2021	80 (25 studies of non-pharmacological interventions)	Included—Only non-pharmacological interventions analyzed
Saritas, S. [46]	A systematic review	Literature searches: Cochrane, CINAHL, PsycINFO, PubMed, EMBA Type of studies: Quasi-experimental, experimental, RCTs. Time: October 2013 and March 2020	13	Included—Without melatonin study
Qin, M. [47]	A systematic review and meta-analysis	Literature searches: PubMed, Embase, MEDLINE, Cochrane Library. Type of studies: RCTs, CBA, and cohort trials. Time: up to September 2021	6	Included
Chen, T-J. [48]	A systematic review and network meta-analysis.	Literature searches: PubMed, EMBASE, CINAHL, Cochrane CENTRAL, ProQuest Dissertations and Theses A&I. Type of studies: RCTs. Time: from the inception to December 2021	29	Included
Liu, J. [49]	A systematic review and meta-analysis	Literature searches: China National Knowledge Infrastructure Database, Excerpta Medica database, PubMed, Cochrane Central Register of Controlled Trials, Wan Fang, Cumulative Index of Nursing and Allied Health Literature. Type of studies: RCTs. Time: from January 2012 to December 2021.	n/d	Excluded—Protocol
Bohart, S. [50]	A systematic review and meta-analysis	Literature searches: MEDLINE, EMBASE, Cochrane Central Register of Controlled Trials, CINAHL, PsycINFO, and Web of Science, hand searched the reference lists of relevant reviews and original trials and searched for unpublished and ongoing studies, and grey literature in Opengrey.eu, and ClinicalTrial.gov. Type of studies: RCTs. Time: n/d	9	Excluded—None of the included studies assessed the number of coma- and delirium-free days in ICU.
Xu, H. [51]	Systematic review and meta-analysis	Literature searches: PubMed, MEDLINE, Cochrane Library, Chinese National Knowledge Infrastructure (CNKI), China Biology Medicine Disc (CBMD), Wanfang Database, and Western Biomedical Journal Database. Type of studies: RCTs. Time: from the establishment to 28 June 2021	7	Included

RCTs—Randomized controlled trial; CCTs—Controlled clinical trial; CBA—Before-and-after studies; PHE—Phenomenological; n/d—no data.

**Table 3:** Critical appraisal results for included studies using the URARI.

Study	Q1	Q2	Q3	Q4	Q5	Q6	Q7	Q8	Q9	Q10	Q11
Litton, E. [29]	Y	Y	Y	Y	Y	Y	Y	Y	Y	Y	n/a
Bannon, L. [31]	Y	Y	Y	Y	Y	Y	Y	Y	Y	Y	Y
Luther, R. [32]	Y	Y	Y	Y	Y	Y	Y	Y	Y	Y	Y
Locihová, H. [33]	Y	Y	Y	Y	U	U	U	Y	Y	Y	Y
Nassar, A.P. [34]	U	Y	Y	Y	Y	Y	Y	Y	Y	Y	n/a
Kang, J. [35]	Y	Y	Y	Y	Y	Y	Y	Y	Y	n/a	n/a
Herling, S.F. [36]	Y	Y	Y	Y	Y	Y	Y	Y	Y	Y	Y
Deng, L.XX [38]	Y	Y	Y	Y	Y	Y	Y	Y	Y	Y	Y
Liang, S. [41]	Y	Y	Y	Y	Y	Y	Y	Y	Y	Y	Y
Burry, L.D. [45]	Y	Y	Y	Y	Y	Y	Y	Y	Y	Y	n/a
Saritas, S. [46]	Y	Y	Y	Y	Y	U	Y	n/a	N	n/a	n/a
Qin, M. [47]	Y	Y	Y	Y	Y	Y	Y	Y	Y	n/a	n/a
Chen, T.J. [48]	Y	Y	Y	Y	Y	Y	Y	Y	Y	Y	n/a
Xu, C. [51]	U	Y	Y	Y	Y	Y	Y	Y	Y	n/a	Y

Y—Yes, N—No, U—Unclear, n/a—not applicable Q1: Was the review question clearly and explicitly stated? Q2: Were the inclusion criteria appropriate for the review question? Q3: Was the search strategy appropriate? Q4: Were the sources and resources used to search for studies adequate? Q5: Were the criteria for appraising studies appropriate? Q6: Was the critical appraisal independently conducted by two or more reviewers? Q7: Were there methods to minimize errors in data extraction? Q8: Were the methods used to combine studies appropriate? Q9: Was the likelihood of publication bias assessed? Q10: Were recommendations for policy and/or practice supported by the reported data? Q11: Were the specific directives for new research appropriate?

**Table 4:** Tabular presentation of qualitative findings of the umbrella review.

Author (First)	Aim	Participants	Interventions	Results	Findings
Litton, E. [30]	To assess the efficacy of earplugs as an ICU strategy for reducing delirium	Adult patients admitted to a critical care environment.	Earplugs—as an isolated intervention (3 studies). Earplugs—as a part of a bundle with eye shades alone (2 studies) or earplugs, eye shades, and additional sleep noise abatement strategies (4 studies).	Earplug placement = RR of 0.59 (95% CI, 0.44–0.78). Hospital mortality: earplug placement was associated with an RR of 0.77 (95% CI, 0.54–1.11).	Earplugs in patients admitted to the ICU, either isolation or as part of a bundle of sleep hygiene improvement, is associated with a significant reduction in risk of delirium.
Luther, R. [32]	To understand whether implementation of chronotherapy within the critical care setting can reduce the prevalence of delirium	Adult patients (18+ years). Critical care settings.	DLA—Controlled dynamic light application; BLT—Bright light therapy; MINI 1—Multi-component non-pharmacological interventions: reduction of lighting and noise; MINI 2—frequent patient orientation, use of music, ear plugs/eye shades, reduction in noise, and use of natural light/dimmed lighting in evening.	DLA: Delirium occurred in 137 of 361 (38%) vs. 123 of 373 (33%) control. BLT: Reductions in delirium occurrence in the groups receiving BLT (collectively 2/16 BLT versus 10/17 control). MINI: Delirium occurred 55 of 167 (33%) pre-intervention (MINI 1) vs. 24 of 171 (14%) post-intervention (*p* < 0.001). Duration of delirium reduced from 3–4 days pre vs. 1–2 days post (*p* = 0.021). Mean sleep efficiency index and increased sleep quality increased. Patients with high sleep efficiency index scores demonstrated significantly reduced risk of delirium. MINI2: Delirium occurred in 10 of 81 (12%) vs. 25 of 79 (31.25%) control (*p* < 0.006). Duration of delirium was also significantly reduced.	Chronotherapy can reduce the incidence of delirium within critical care.
Locihová, H. [33]	To comment on the effectiveness of selected non-pharmacological interventions and to provide a basis for discussion of whether these measures may have an impact upon the improvement of the short-term (reduction of delirium, shortening of hospitalization time) and long-term outcomes.	Patients in ICUs.	Plugs; Eye mask; Plugs and eye mask; Plugs, mask, and music	Earplugs: Cox regression analysis revealed a reduction in the risk of early development of delirium and confusion by 53%. Earplugs + eye mask + relaxing music: confirmed a statistically significant reduction in the delirium incidence of the investigated interventions: pre-phase: (22%), cf. post-phase (49%; OR: 0.46, 95% CI: 0.23–0.89, *p* = 0.02) and confirmed a statistically significant difference in the occurrence of the daily delirium-free status in patients in the pre-phase (43%) cf. post-phase (48%; OR: 1.64, 95% CI: 1.04–2.58, *p* = 0.03). Earplugs + eye mask: confirmed a statistically significant reduction in the incidence of postoperative disorientation in the intervention group (control group 14%, cf. intervention group 0%, *p* = 0.01).	The examined interventions reduce the incidence of delirium significantly.
Nassar Junior, A.P. [34]	To synthesize data on outcomes related to patients, family members, and ICU professionals by comparing flexible vs. restrictive visiting policies in ICUs.	ICU-patients, family members, ICU-professionals.	Flexible visiting policies.	Two studies evaluated the frequency of delirium (354 patients). The flexible visiting policy was associated with a reduced frequency of delirium (OR, 0.39; 95% CI, 0.22–0.69; I2 = 0%).	Flexible ICU visiting hours have the potential to reduce delirium.
Kang, J. [35]	To examine the effect of nonpharmacological interventions that are used in the prevention of ICU delirium.	Adult patients (>18 years) admitted to an ICU of various types (ICU, MICU, SICU in five studies (14.3%), MICU and SICU in cardiac ICU, traumatic, and cardiac care unit).	MLT—multicomponent interventions; PEI—physical environment interventions; DIS—daily interruption of sedation, exercise; PE—patient education; AWS—automatic warning system; CHI—cerebral hemodynamics improvement; FP—family participation; SR—sedation reduction.	The effect sizes of non-pharmacological interventions for onset of delirium and duration of delirium were statistically significant. The effect sizes for length of ICU stay and ICU mortality were not statistically significant. The effect size in relation to the occurrence of delirium was statistically significant only for MLT.	MLTs significantly reduced the occurrence of delirium but did not significantly shorten the duration of delirium.
Herling, S.F. [36]	To assess existing evidence for the effect of preventive interventions on ICU delirium, in-hospital mortality, the number of delirium-, coma-, and ventilator-free days, length of stay in the ICU and cognitive impairment.	Adult medical or surgical ICU patients	Physical or cognitive therapy interventions or both, environmental interventions with changes in light or sound/hearing (earplugs), and nursing care intervention.	Physical and cognitive therapy versus standard care: no effect of the intervention; Early mobilization and occupational therapy: positive effects of the intervention time on return to independent function and ventilator-free days and duration of delirium within the first 28 days of hospital stay. Environmental intervention versus standard care: no significant difference between groups. Preventive nursing care interventions: no effect on the event rate of ICU delirium, in-hospital mortality, and on length of ICU stay.	Physical, cognitive, and occupational therapy interventions may have a potential for preventing or reducing the duration of delirium.
Bannon, L. [31]	To evaluate the effect of non-pharmacological interventions versus standard care on incidence and duration of delirium in critically ill patients.	ICU patient populations including medical surgical and mixed medical and surgical.	Physical and physical with occupational therapy; bright light therapy; range of motion exercises; earplugs; multicomponent orientation and cognitive stimulation protocol; multicomponent occupational therapy including positioning, cognitive training, and relative involvement; a mirrors intervention; multicomponent targeting risk factors for delirium; protocolized weaning and daily sedation interruption; reorientation using family voice; and paired awakening and breathing.	Incidence of delirium: BLT and individual interventions showed no significant effect between groups. Duration of delirium: MLT physical therapy and various individual interventions showed no significance. Family voice reorientation showed a beneficial effect.	Only family voice reorientation showed a beneficial effect on delirium duration.
Deng, L. [38]	To compare non-pharmacological interventions in their ability to prevent delirium in critically ill patients.	Adult patients (>18 years) admitted to ICU of any type.	CHI—cerebral hemodynamic improving; PEI—physical environment intervention; SR—sedation reduction; FP—family participation; EP—exercise program; MLT—multicomponent interventions; UC—usual care.	The most effective intervention in reducing the incidence of delirium was: FP (94%), EP (74%), MLT (68%), CHI (58%), PEI (26%), and SR (18%). In terms of reduction in in-hospital mortality, EP ranked highest (97.2%), followed by: MLT (73.2%), CHI (35.8%), PEI (34.8%), and SR (31.8%). Although not statistically significant, MLT ranked first in both reducing the number of days of delirium (78.6%) and reducing the length of stay in the intensive care unit (71.2%).	MLT are promising; FP has also shown promise as an intervention in reducing the incidence of delirium (still needs further study).
Liang, S. [41]	To determine the effects of non-pharmacological interventions on preventing delirium and improving critically ill patients’ clinical, psychological, and family outcomes.	Adult patients (>18 years) admitted to an ICU of various types (surgical, medical, trauma, or cardiac ICUs or a high-dependency unit). Studies involving ICU patients with a history of neurological disorders were excluded.	EM—early mobilization; FP—family participation; PE—patient education; M—music; SP—sleep promotion; PEI—physical environment intervention; MLT—multicomponent interventions; UC—usual care.	MLT had a higher OR than single component interventions. EM in the combined analyses showed a statistically significant effect on reducing the incidence of delirium and duration. FP-analysis pooled showed a statistically significant effect on reducing the incidence of delirium. Additionally, pooled analysis of three of these studies showed a positive effect on LOS in the intensive care unit. There was a statistically significant effect of music on reducing the incidence of delirium (M). Pooled analysis showed that PE caused a statistically significant reduction in the incidence of delirium. The use of earplugs reduced the risk of delirium or disorientation by 53% (SP).	MLT should be a priority for the prevention of delirium in the ICU in clinical practice; FP and EM can be effective non-pharmacological methods for the prevention of delirium in ICU patients.
Burry, LD. [45]	To compare the effects of prevention interventions on delirium occurrence in critically ill adults.	Critically ill adults (≥16 years of age in an ICU of any type or high-acuity unit).	Occupational therapy, Early physical therapy daily, Early physical therapy + cognitive exercises, Music, Eye mask + ear plugs + routine night care, Family intervention, Multi-component strategies, Mirrors, Noise reduction, refurbished rooms with suspended ceiling and low frequency sound absorption, Family intervention, orientation training/supervision (memory guidance), therapeutic engagement (cognitive stimulation) and sensory control (e.g., glasses and hearing aids), Delirium prevention protocol including screening for delirium risk factors, subsequent cognitive assessment and orientation, environmental management and therapeutic intervention, Interprofessional early mobilization protocol, Bright light therapy, Standard post-stroke care, therapeutic activities twice daily based on the Hospital Elder Life Program and assessment of anticholinergic burden and medication risk, ABCDE bundle daily.	Pairwise comparisons for single or multicomponent non-pharmacological interventions found no differences compared to standard care for ICU or hospital length of stay, except for mobilization with occupational or physical therapists compared to standard care.	Single and multicomponent non-pharmacological interventions did not connect to any evidence networks to allow for ranking and comparisons as planned; pairwise comparisons did not detect differences compared to standard care.
Saritas, S. [46]	To prepare a systematic review with articles that tested the effectiveness of non-pharmacological interventions towards preventing delirium at adult intensive care units.	Patients hospitalized at secondary or tertiary institutions’ adult ICUs.	MLT—multicomponent, PE—patient education, HI—hormone intervention, PEI—physical environment intervention, TI—therapeutic intervention, APS—automated preventive system, QDS—quitting daily sedation and exercise.	All interventions were effective. The multicomponent intervention was statistically significantly effective in terms of reducing/preventing delirium.	The interventions had important effects regarding delirium management, but only the MLT application was significant
Qin, M. [47]	To evaluate the effects of family intervention on the incidence and duration of delirium, length of ICU stay, and duration of ventilation in ICU patients.	Adult ICU patients.	Orientation—memory clues delivered by family members, family members’ voices, flexible visitation, or standard family visitation.	Family intervention was associated with a 24% lower risk of delirium. Family intervention reduced the number of delirium days.	Family intervention was associated with a lower risk of delirium and fewer delirium days, but it did not affect the length of ICU stay, the duration of ventilation, or patient mortality.
Xu, H. [51]	Impact of cognitive exercise on the incidence of delirium in ICU inpatients.	Adult patients with delirium in the ICU.	Cognitive exercise	The duration of delirium in the treatment group and routine group was significantly different (Z = 3.24, MD = −1.99, 95% CI: −3.20, −0.79, *p* = 0.001). That cognitive exercise significantly shortened the length of hospital stay in ICU patients with delirium (Z = 10.84, MD = −2.10, 95% CI: −2.48, −1.72, *p* < 0.00001).	Cognitive exercises can reduce the incidence and duration of delirium in ICU inpatients and shorten the length of hospitalization.
Chen, T-J. [48]	To compare the effects of non-pharmacological interventions by combining direct and indirect evidence of the incidence and duration of delirium in intensive care units.	Adults (age ≥ 18 years) in ICU.	EC—environment control; CA—clinical adjustment; PA—physical activity; HE—health education; Multi_A, B, C, D—multicomponent A, B, C, D; LT = light therapy; FM = fluid management; EM—early mobilization, FV—family visit, EE—eye mask and earplugs, EEM—eye mask, earplugs, and melatonin, PHE—preoperative health education.	Multi_A significantly reduced delirium incidence risk compared to routine care (OR = 0.12, 95% CI = 0.02 to 0.83) and was ranked best based on the findings of SUCRA (87.4%).	Multicomponent non-pharmacological interventions are the most effective intervention for ICU delirium prevention but not ICU delirium duration.

## Data Availability

The authors declare that the data of this research are available from the correspondence author upon request.

## References

[1] Hshieh T.T., Inouye S.K., Oh E.S. (2018). Delirium in the Elderly. Psychiatr. Clin. N. Am..

[2] Volland J., Fisher A., Drexler D. (2020). Preventing and identifying hospital-acquired delirium. Nursing.

[3] Wilson J.E., Mart M.F., Cunningham C., Shehabi Y., Girard T.D., MacLullich A.M.J., Slooter A.J.C., Ely E.W. (2020). Delirium. Nat. Rev. Dis. Prim..

[4] Kotfis K., Marra A., Ely E.W. (2018). ICU delirium—A diagnostic and therapeutic challenge in the intensive care unit. Anaesthesiol. Intensiv. Ther..

[5] Ely E.W., Shintani A., Truman B., Speroff T., Gordon S.M., Harrell F.E., Inouye S.K., Bernard G.R., Dittus R.S. (2004). Delirium as a Predictor of Mortality in Mechanically Ventilated Patients in the Intensive Care Unit. J. Am. Med. Assoc..

[6] Hayhurst C.J., Pandharipande P.P., Hughes C.G. (2016). Intensive Care Unit Delirium: A Review of Diagnosis, Prevention, and Treatment. Anesthesiology.

[7] Inouye S.K., Bogardus S.T., Baker D.I., Leo-Summers L., Cooney L.M. (2000). The hospital elder life program: A model of care to prevent cognitive and functional decline in older hospitalized patients. J. Am. Geriatr. Soc..

[8] Tran N.N., Hoang T.P.N., Ho T.K.T. (2021). Diagnosis and risk factors for delirium in elderly patients in the emergency rooms and intensive care unit of the national geriatric hospital emergency department: A cross-sectional observational study. Int. J. Gen. Med..

[9] Ozga D., Krupa S., Witt P., Mędrzycka-Dabrowska W. (2020). Nursing interventions to prevent delirium in critically ill patients in the intensive care unit during the covid19 pandemic—narrative overview. Healthcare.

[10] McKenzie J., Joy A. (2020). Family intervention improves outcomes for patients with delirium: Systematic review and meta-analysis. Australas. J. Ageing.

[11] Hipp D.M., Ely E.W. (2012). Pharmacological and Nonpharmacological Management of Delirium in Critically Ill Patients. Neurotherapeutics.

[12] Pun B.T., Ely E.W. (2007). The importance of diagnosing and managing ICU delirium. Chest.

[13] Krupa S., Ozga D. (2019). Review of the Literature on the Occurrence of Delirium after Veno-Venous and Veno-Arterial Extracorporeal Membrane Oxygenation: A Systematic Review. Dement. Geriatr. Cogn. Dis. Extra.

[14] Cascella M., Fiore M., Leone S., Carbone D., Di Napoli R. (2019). Current controversies and future perspectives on treatment of intensive care unit delirium in adults. World J. Crit. Care Med..

[15] Devlin J.W., Skrobik Y., Gélinas C., Needham D.M., Slooter A.J.C., Pandharipande P.P., Watson P.L., Weinhouse G.L., Nunnally M.E., Rochwerg B. (2018). Clinical Practice Guidelines for the Prevention and Management of Pain, Agitation/Sedation, Delirium, Immobility, and Sleep Disruption in Adult Patients in the ICU. Crit. Care Med..

[16] Blodgett T.J., Blodgett N.P. (2021). Melatonin and melatonin-receptor agonists to prevent delirium in hospitalized older adults: An umbrella review. Geriatr. Nurs..

[17] Khaing K., Nair B.R. (2021). Melatonin for delirium prevention in hospitalized patients: A systematic review and meta-analysis. J. Psychiatr. Res..

[18] Ng K.T., Teoh W.Y., Khor A.J. (2020). The effect of melatonin on delirium in hospitalised patients: A systematic review and meta-analyses with trial sequential analysis. J. Clin. Anesth..

[19] Osiezagha K., Ali S., Freeman C., Barker N.C., Jabeen S., Maitra S., Olagbemiro Y., Richie W., Bailey T.K. (2013). Thiamine deficiency and delirium. Innov. Clin. Neurosci..

[20] McKenzie C.A., Page V.J., Strain W.D., Blackwood B., Ostermann M., Taylor D., Peter E., Spronk P.E., McAuley D.F. (2020). Parenteral thiamine for prevention and treatment of delirium in critically ill adults: A systematic review protocol. Syst. Rev..

[21] Sedhai Y.R., Shrestha D.B., Budhathoki P., Jha V., Mandal S.K., Karki S., Baniya R., Cable C.A., Kashiouris M.G. (2021). Effect of thiamine supplementation in critically ill patients: A systematic review and meta-analysis. J. Crit. Care.

[22] Lange S., Mędrzycka-Dąbrowska W., Friganovic A., Oomen B., Krupa S. (2021). Delirium in critical illness patients and the potential role of thiamine therapy in prevention and treatment: Findings from a scoping review with implications for evidence-based practice. Int. J. Environ. Res. Public Health.

[23] Martinez F., Tobar C., Hill N. (2015). Preventing delirium: Should non-pharmacological, multicomponent interventions be used? A systematic review and meta-analysis of the literature. Age Ageing.

[24] Burton J.K., E Craig L., Yong S.Q., Siddiqi N., A Teale E., Woodhouse R., Barugh A.J., Shepherd A.M., Brunton A., Freeman S.C. (2021). Non-pharmacological interventions for preventing delirium in hospitalised non-ICU patients. Cochrane Database Syst. Rev..

[25] Rivosecchi R.M., Smithburger P.L., Svec S., Campbell S., Kane-Gill S.L. (2015). Nonpharmacological interventions to prevent delirium: An evidence-based systematic review. Crit. Care Nurse.

[26] Papatheodorou S.I., Evangelou E. (2022). Umbrella Reviews: What They Are and Why We Need Them. Methods Mol. Biol..

[27] Papatheodorou S.I. (2019). Umbrella reviews: What they are and why we need them. Eur. J. Epidemiol..

[28] Aromataris E., Fernandez R., Godfrey C., Holly C., Khalil H., Tungpunkom P. (2014). The Joanna Briggs Institute Reviewers’ Manual 2014 Methodology for JBI Umbrella Reviews.

[29] Zhang H., Lu Y., Liu M., Zou Z., Wang L., Xu F.-Y., Shi X.-Y. (2013). Strategies for prevention of postoperative delirium: A systematic review and meta-analysis of randomized trials. Crit. Care.

[30] Litton E., Carnegie V., Elliott R., Webb S.A.R. (2016). The Efficacy of Earplugs as a Sleep Hygiene Strategy for Reducing Delirium in the ICU: A Systematic Review and Meta-Analysis. Crit. Care Med..

[31] Bannon L., McGaughey J., Verghis R., Clarke M., McAuley D.F., Blackwood B. (2019). The effectiveness of non-pharmacological interventions in reducing the incidence and duration of delirium in critically ill patients: A systematic review and meta-analysis. Intensiv. Care Med..

[32] Luther R., McLeod A. (2018). The effect of chronotherapy on delirium in critical care—A systematic review. Nurs. Crit. Care.

[33] Locihová H., Axmann K., Padyšáková H., Fejfar J. (2018). Effect of the use of earplugs and eye mask on the quality of sleep in intensive care patients: A systematic review. J. Sleep Res..

[34] Nassar A.P., Besen B.A.M.P., Robinson C.C., Falavigna M., Teixeira C., Rosa R.G. (2018). Flexible versus restrictive visiting policies in ICUs: A systematic review and meta-analysis. Crit. Care Med..

[35] Kang J., Lee M., Ko H., Kim S., Yun S., Jeong Y., Ch Y. (2018). Effect of nonpharmacological interventions for the prevention of delirium in the intensive care unit: A systematic review and meta-analysis. J. Crit. Care.

[36] Herling S.F., Greve I.E., Vasilevskis E.E., Egerod I., Mortensen C.B., Møller A.M., Svenningsen H., Thomsen T. (2018). Interventions for preventing intensive care unit delirium in adults. Cochrane Database Syst. Rev..

[37] Janssen T.L., Alberts A.R., Hooft L., Mattace-Raso F.U.S., Mosk C.A., Van Der Laan L. (2019). Prevention of postoperative delirium in elderly patients planned for elective surgery: Systematic review and meta-analysis. Clin. Interv. Aging.

[38] Deng L.-X., Cao L., Zhang L.-N., Peng X.-B., Zhang L. (2020). Non-pharmacological interventions to reduce the incidence and duration of delirium in critically ill patients: A systematic review and network meta-analysis. J. Crit. Care.

[39] León-Salas B., Trujillo-Martín M.M., Del Castillo L.P.M., García-García J., Pérez-Ros P., Rivas-Ruiz F., Serrano-Aguilar P. (2020). Multicomponent Interventions for the Prevention of Delirium in Hospitalized Older People: A Meta-Analysis. J. Am. Geriatr. Soc..

[40] Ludolph P., Msc J.S., Kunzler A.M., Rösch R., Geschke K., Vahl C.F., Lieb K. (2020). Non-Pharmacologic Multicomponent Interventions Preventing Delirium in Hospitalized People. J. Am. Geriatr. Soc..

[41] Liang S., Chau J.P.C., Lo S.H.S., Zhao J., Choi K.C. (2021). Effects of nonpharmacological delirium-prevention interventions on critically ill patients’ clinical, psychological, and family outcomes: A systematic review and meta-analysis. Aust. Crit. Care.

[42] Ekeozor C.U., Jeyaruban D., Lasserson D. (2021). Where should patients with or at risk of delirium be treated in an acute care system? Comparing the rates of delirium in patients receiving usual care vs alternative care: A systematic review and meta-analysis. Int. J. Clin. Pract..

[43] de Foubert M., Cummins H., McCullagh R., Brueton V., Naughton C. (2021). Systematic review of interventions targeting fundamental care to reduce hospital-associated decline in older patients. J. Adv. Nurs..

[44] Lee Y., Lee J., Kim J., Jung Y. (2021). Non-pharmacological nursing interventions for prevention and treatment of delirium in hospitalized adult patients: Systematic review of randomized controlled trials. Int. J. Environ. Res. Public Health.

[45] Burry L.D., Cheng W., Williamson D.R., Adhikari N.K., Egerod I., Kanji S., Martin C.M., Hutton B., Rose L. (2021). Pharmacological and non-pharmacological interventions to prevent delirium in critically ill patients: A systematic review and network meta-analysis. Intensiv. Care Med..

[46] Saritas S., Tarlaci S. (2021). A Systematic Review of Non-Pharmacological Interventions to Prevent Delirium at Intensive Care Units. Psychiatry Behav. Sci..

[47] Qin M., Gao Y., Guo S., Lu X., Zhu H., Li Y. (2022). Family intervention for delirium for patients in the intensive care unit: A systematic meta-analysis. J. Clin. Neurosci..

[48] Chen T.J., Traynor V., Wang A.Y., Shih C.Y., Tu M.C., Chuang C.H., Chiu H.-Y., Chang H.-C. (2022). Comparative Effectiveness of Non-Pharmacological Interventions for Preventing Delirium in Critically Ill Adults: A Systematic Review and Network Meta-Analysis. Int. J. Nurs. Stud..

[49] Liu J., Wang J. (2022). Efficacy of EWINDOW for prevention of delirium at intensive care units: A protocol for systematic review and meta-analysis. Medicine.

[50] Bohart S., Møller A.M., Andreasen A.S., Waldau T., Lamprecht C., Thomsen. T. (2022). Effect of Patient and Family Centred Care interventions for adult intensive care unit patients and their families: A systematic review and meta-analysis. Intensiv. Crit. Care Nurs..

[51] Xu C., Chen Z., Zhang L., Guo H. (2022). Systematic review and meta-analysis on the incidence of delirium in intensive care unit inpatients after cognitive exercise intervention. Ann. Palliat. Med..

[52] Aromataris E., Munn Z. (2020). JBI Manual for Evidence Synthesis.

[53] Pezzullo L., Streatfeild J., Hickson J., Teodorczuk A., Agar M.R., Caplan G.A. (2019). Economic impact of delirium in Australia: A cost of illness study. BMJ Open.

[54] Cavallazzi R., Saad M., Marik P.E. (2012). Delirium in the ICU: An overview. Ann. Intensiv. Care.

[55] Eckstein C., Burkhardt H. (2019). Multicomponent, nonpharmacological delirium interventions for older inpatients: A scoping review. Z. Gerontol. Geriatr..

[56] Sahawneh F., Boss L. (2021). Non-pharmacologic interventions for the prevention of delirium in the intensive care unit: An integrative review. Nurs. Crit. Care.

[57] Wright S.E., Thomas K., Watson G., Baker C., Bryant A., Chadwick T.J., Jing Shen J., Wood R., Wilkinson J., Mansfield L. (2018). Intensive versus standard physical rehabilitation therapy in the critically ill (EPICC): A multicentre, parallel-group, randomised controlled trial. Thorax.

[58] Doiron K., Hoffmann T., Beller E. (2018). Early intervention (mobilization or active exercise) for critically ill adults in the intensive care unit (Review). Cochrane Database Syst. Rev..

[59] Schweickert W.D., Pohlman M.C., Pohlman A.S., Nigos C., Pawlik A.J., Esbrook C.L., Spears L., Miller M., Franczyk M., Deprizio D. (2009). Early physical and occupational therapy in mechanically ventilated, critically ill patients: A randomised controlled trial. Lancet.

[60] Morris P.E., Berry M.J., Files D.C., Thompson J.C., Hauser J., Flores L., Dhar S., Chmelo E., Lovato J., Case L.D. (2016). Standardized rehabilitation and hospital length of stay among patients with acute respiratory failure a randomized clinical trial. JAMA—J. Am. Med. Assoc..

[61] Engwall M., Fridh I., Johansson L., Bergbom I., Lindahl B. (2015). Lighting, sleep and circadian rhythm: An intervention study in the intensive care unit. Intensiv. Crit. Care Nurs..

[62] Guo Y., Sun L., Li L., Jia P., Zhang J., Jiang H., Jiang W. (2016). Impact of multicomponent, nonpharmacologic interventions on perioperative cortisol and melatonin levels and postoperative delirium in elderly oral cancer patients. Arch. Gerontol. Geriatr..

[63] Ono H., Taguchi T., Kido Y., Fujino Y., Doki Y. (2011). The usefulness of bright light therapy for patients after oesophagectomy. Intensiv. Crit. Care Nurs..

[64] Kleinpell R., Zimmerman J., Vermoch K.L., Harmon L.A., Vondracek H., Hamilton R., Hanson B., Hwang D.Y. (2019). Promoting Family Engagement in the ICU: Experience from a National Collaborative of 63 ICUs. Crit. Care Med..

[65] Page M.J., McKenzie J.E., Bossuyt P.M., Boutron I., Hoffmann T.C., Mulrow C.D., Shamseer L., Tetzlaff J.M., Akl E.A., Brennan S.E. (2021). The PRISMA 2020 statement: An updated guideline for reporting systematic reviews. BMJ.

[66] Pabón-Martínez B.A., Rodríguez-Pulido L.I., Henao-Castaño A.M. (2022). The family in preventing delirium in the intensive care unit: Scoping review. Enferm. Intensiva.

[67] Ely E.W. (2017). The ABCDEF bundle: Science and philosophy of how ICU liberation serves patients and families. Crit. Care Med..

